# Probiotics Affect One‐Carbon Metabolites and Catecholamines in a Genetic Rat Model of Depression

**DOI:** 10.1002/mnfr.201701070

**Published:** 2018-03-13

**Authors:** Sandra Tillmann, Hussain M. Awwad, Amanda R. Eskelund, Giulia Treccani, Juergen Geisel, Gregers Wegener, Rima Obeid

**Affiliations:** ^1^ Translational Neuropsychiatry Unit Department of Clinical Medicine Aarhus University Risskov Denmark; ^2^ Saarland University Hospital Department of Clinical Chemistry and Laboratory Medicine Homburg/Saar Germany; ^3^ Aarhus Institute of Advanced Studies Aarhus University Aarhus C Denmark

**Keywords:** depression, dopamine, gut–brain axis, probiotics, S‐adenosylmethionine

## Abstract

**Scope:**

Probiotics may influence one‐carbon (C1) metabolism, neurotransmitters, liver function markers, or behavior.

**Methods and results:**

Male adult Flinders Sensitive Line rats (model of depression, FSL; *n* = 22) received *Lactobacillus helveticus* R0052 and *Bifidobacterium longum* R0175 (10^9^ or 10^10^ colony‐forming units per day) or vehicle for 10 weeks. The controls, Flinders Resistant Line rats (FRL, *n* = 8), only received vehicle. C1‐related metabolites were measured in plasma, urine, and different tissues. Monoamine concentrations were measured in plasma, hippocampus, and prefrontal cortex. Vehicle‐treated FSL rats had higher plasma concentrations of betaine, choline, and dimethylglycine, but lower plasma homocysteine and liver S‐adenosylmethionine (SAM) than FRLs. FSL rats receiving high‐dose probiotics had lower plasma betaine and higher liver SAM compared to vehicle‐treated FSL rats. FSLs had higher concentrations of norepinephrine, dopamine, and serotonin than FRLs across various brain regions. Probiotics decreased plasma dopamine in FSLs in a dose‐dependent manner. There were no detectable changes in liver function markers or behavior.

**Conclusions:**

Probiotics reduced the flow of methyl groups via betaine, increased liver SAM, and decreased plasma dopamine and norepinephrine. Since these changes in methylation and catecholamine pathways are known to be involved in several diseases, future investigation of the effect of probiotics is warranted.

## 1 Introduction

The intestinal microbiota plays a pivotal role in human health, and alterations in its composition have been associated with gastrointestinal, hepatic, metabolic, and central nervous system‐related diseases.^1^, ^2^ Probiotics have been used to restore the gut microbiota and have shown promising efficacy in gastrointestinal diseases^3^ and alcohol‐related liver damage,^2^, ^4^ while the influence on depression in humans is controversial.^5^, ^6^, ^7^ The underlying mechanisms of bacteria–host interaction remain elusive. However, it has been shown that the bacteria produce biologically active signaling molecules,^8^, ^9^ which may cross the intestinal barrier to reach the host circulation and thereby affect metabolic pathways.^10^ The synthesis of neurotransmitters may be a direct or indirect pathway to induce changes in the central nervous system, as probiotics have been shown to produce neuroactive compounds, including gamma‐aminobutyric acid, norepinephrine, serotonin, dopamine, and acetylcholine.^8^

Moreover, several lactic acid bacterial strains^9^, ^11^ are in vitro sources of S‐adenosylmethionine (SAM), a ubiquitous methyl donor in mammalian cells required by over 100 cellular methyl transfer reactions, including DNA methylation and synthesis of neurotransmitters or phosphatidylcholine.^12^, ^13^ In humans, SAM is formed mainly in the liver from various dietary methyl donors such as methionine, folate, betaine, or choline.^14^ SAM is required for a variety of methyltransferases such as catechol‐O‐methyltransferase (COMT) and phenylethanolamine *N*‐methyltransferase (PNMT). Deficiency of methyl donors such as folate or choline may cause fatty liver^15^ and disorders of the central nervous system, including depression.^16^, ^17^ Direct supplementation of SAM shows generally protective effects on the liver and central nervous system.^18^, ^19^ However, it is not known whether oral supplementation of lactic acid bacteria can increase SAM in vivo or influence monoamine neurotransmitters in the host.

Intervention with probiotics may affect nutrient bioavailability, metabolism, or utilization in the host, as gut bacteria and host seem to compete for nutrients. We hypothesized that the exposure to probiotics (*Lactobacillus helveticus* R0052 and *Bifidobacterium longum* R0175) at two different doses can alter one‐carbon (C1) metabolism, monoamine neurotransmitters, liver function, and depressive‐like behavior in a rat model of depression.

## 2 Experimental Section

### 2.1 Animals

Healthy male adult Flinders Sensitive Line (FSL, *n* = 22) and Flinders Resistant Line (FRL, *n* = 8) rats were obtained from the breeding colony maintained at Aarhus University, Denmark (originally derived from the colony at the University of North Carolina, USA). The FSL rat is a validated genetic animal model of depression. FRL rats are used as controls to ascertain the depressive‐like phenotype of FSL rats.^20^, ^21^, ^22^ Animals were allowed to acclimatize for 2 weeks before intervention start and were then weighed once a week over the 10‐week intervention period. No additional handling took place other than that used as part of the probiotic administration method described under 2.2. Three days before the start of the intervention, FSL rats were (mean ± SD) 10.2 ± 0.4 weeks old and weighed 285 ± 32 g. FRL rats were 9.4 ± 0.9 weeks old and weighed 319 ± 21 g. FSL rats were significantly older (*p* = 0.004) and weighed less (*p* = 0.030) than FRL rats. The lower weight is part of the characteristic phenotype of the FSL animals^21^; the age difference can be considered negligible. Animals of the same strain and treatment group were pair‐housed in standard cages (Cage 1291H Eurostandard Type III H, 425 × 266 × 185 mm, Tecniplast, Italy) at 20 ± 2 °C and 60 ± 5% relative humidity on a reversed 12 h light/dark cycle (lights on at 2 p.m.), which was introduced in 3‐h increments right before the 2‐week acclimatization phase. Cages were changed once a week. Tap water and chow diet were available to all animals ad libitum, along with access to a tunnel shelter, nesting material, and a wooden stick. Rats were fed a standard chow diet (#1324 Altromin, Brogaarden ApS, Lynge, Denmark; Supporting Information Table 1). All animal experiments were conducted at Translational Neuropsychiatry Unit, Department of Clinical Medicine, Aarhus University, Denmark. All experiments were approved by the Danish Animal Experiments Inspectorate prior to initiation of the experiments (approval number: 2012‐15‐2934‐00254) and were conducted in accordance with the European Communities Council Directive.

### 2.2 Experimental Design and Intervention

FSL rats were randomly assigned to one of three groups: vehicle (*n* = 7); probiotics at a low dose of 10^9^ colony‐forming units per day (CFU d^−1^) (*n* = 7); or probiotics at a 10‐fold higher dose of 10^10^ CFU d^−1^ (*n* = 8). FRL rats (*n* = 8) only received vehicle (Supporting Information Figure 1) to control for the depressive‐like phenotype.

The commercial probiotic formulation Probio'Stick (Lallemand Health Solutions Inc., Montreal, QC, Canada) was used. This product contained a mixture of freeze‐dried lactic acid bacteria (*L. helveticus* R0052 and *B. longum* R0175) and excipients (xylitol, maize‐derived maltodextrin, plum flavor, malic acid). The vehicle formulation was of identical taste and consisted of the same excipients without any active cultures. The probiotic and vehicle solutions were freshly prepared just before administration by dissolving the respective weight of the powder in tap water (vehicle: 6 g in 9 mL water, daily dose per rat: 0.2 g; low‐dose probiotics: 0.4 g in 6 mL water, daily dose per rat: 0.02 g; high‐dose probiotics: 4 g in 6 mL water, daily dose per rat: 0.2 g). Rats received the intervention dose dissolved in a total volume of 0.3 mL administered via syringe‐feeding (i.e., consuming the probiotics directly from a syringe held into their cage)^23^ once daily toward the end of the active phase (2 p.m. ± 1 h) over the 10‐week treatment period. Animal weight as well as water and food intake (per 100 g bodyweight) were recorded throughout the study.

### 2.3 Tissue Collection

Rats were decapitated after 10 weeks of starting the treatment. Decapitation took place between 2 and 5 p.m. over 2 consecutive days and was conducted in a random order. To minimize stress, rats were housed in an adjacent room and were brought singly into the decapitation room by an experimenter free of blood scent. Urine and fecal boli were collected 2 d before decapitation and immediately frozen at −80 °C until further analyses. After decapitation, trunk blood was collected in EDTA‐coated tubes and immediately centrifuged at 3000 × *g* for 10 min. Plasma was aliquoted and stored at −80 °C. Liver tissue (from the left lateral lobe), hippocampi, and prefrontal cortices were quickly removed, snap‐frozen in pre‐cooled isopentane, and stored at −80 °C. Hippocampal and prefrontal cortex tissue was extracted from both hemispheres. To prevent hemispheric bias, half of the SAM, S‐adenosylhomocysteine (SAH), and monoamine measurements were conducted in the left hemisphere and half in the right one.

### 2.4 Sample Processing and Biochemical Measurements

Supporting Information Table 2 shows the types of tissues collected, the preservation method, and the markers measured in the respective tissues. Frozen fecal samples were thawed on ice, weighed, and extracted either in distilled water (10 μL H_2_O mg^−1^ feces) for betaine, choline, and dimethylglycine assays, or in 1 n acetic acid for SAM and SAH assays (10 μL 1 n acetic acid mg^−1^ feces). After centrifugation at 10,000 × *g* for 10 min, the supernatants were used for the assays.

Snap‐frozen livers, hippocampi, and prefrontal cortices were thawed on ice. Liver tissues were washed with cold PBS to remove possible blood contaminations. Tissues were then weighed and homogenized in 1 n acetic acid (10 μL 1 n acetic acid mg^−1^ tissue) using blades. After centrifugation at 10,000 × *g* for 10 min, the supernatants were collected and used for SAM and SAH assays. Aliquots of frozen EDTA plasma and urine samples were thawed and used immediately for measurements of the biomarkers (Supporting Information Table 2).

Frozen brain tissue samples were sonicated (50% power, 4 s, Bandelin Sonopuls UW 2200) in cold 0.2 n perchloric acid (5 μL mg^−1^ tissue) and centrifuged at 10,000 × *g* at 4 °C for 10 min. The supernatant was collected, filtered using Costar Spin‐X (0.22 μm Cellulose Acetate membrane, Corning, NY, USA), and used for measurements of monoamines. For plasma samples, 20 μL perchloric acid was added to 180 μL of plasma, followed by centrifugation and supernatant collection as described above.

The concentrations of C1‐related metabolites were measured at the Department of Clinical Chemistry, Saarland University Hospital, Germany by using established methods on an Acquity Ultra Performance LC system coupled to a MicroMass Quattro Premier XE tandem quadrupole mass spectrometer (UPLC‐MS/MS) (Waters Corporation, Milford, MA, USA). Plasma concentrations of total homocysteine and cystathionine were measured using an in‐house gas‐chromatography mass spectrometry method. The concentrations of betaine, choline, and dimethylglycine were measured in EDTA plasma, urine, and fecal water extracts by using labelled internal standards and acetonitrile precipitation as described previously.^24^ For all urinary markers, urine samples were diluted 1:5 in water before measurement of the metabolites.

Concentrations of SAM and SAH in the acidified extracts of liver, brain, and fecal samples were measured using established methods that depend on the use of labelled isotopes as internal standards (^13^C5‐SAH and ^2^H3‐SAM).^25^ The sample volume of the extracts was chosen to obtain measured concentrations within the range of the standard curve (up to 300 nmol L^−1^ for SAM and 150 nmol L^−1^ for SAH). All markers measured in tissues are expressed as nmol g^−1^ tissue. Plasma liver markers, alanine transaminase (ALT), and aspartate transaminase (AST), were measured using routine automated methods. Supporting Information Table 3 shows information on method performances such as between‐day coefficient of variations and limits of detections.

Concentrations of serotonin (5‐HT), 5‐hydroxyindoleacetic acid (5‐HIAA), dopamine, 3,4‐dihydroxyphenylacetic acid (DOPAC), and norepinephrine were measured in plasma samples and tissue extracts of hippocampus and prefrontal cortex at Translational Neuropsychiatry Unit, Aarhus University, Denmark by using established methods as described elsewhere.^26^ Measurements were conducted using Ultrahigh performance liquid chromatography with electrochemical detection (UHPLC‐ECD, Dionex Ultimate 3000 UHPLC, Thermo Scientific, Rockford, IL, USA). The mobile phase contained 75 mM sodium dihydrogen phosphate monohydrate, 10% acetonitrile, 1.7 mM 1‐octanesulfonic acid, 0.025 mM ethylenediaminetetraacetic acid, and 0.0001% triethylamine (pH = 3). Neurotransmitters were separated on a Kinetex 2.6 μm C18, 150 × 4.6 mm evolution column (with a SecurityGuard Ultra Cartridge and holder (both Phenomenex, Torrance, CA, USA)). The column temperature was 27 °C and a Dionex 6011RS Ultra Analytical Cell (Thermo Scientific, Rockford, IL, USA) was used to detect the compounds. Concentrations of neurotransmitters are expressed as nmol L^−1^ (in plasma) or nmol g^−1^ tissue.

### 2.5 Behavioral Assessment

Six weeks after starting the intervention, standard tests for cognition, anxiety, locomotion, and depression were conducted over the following 3 weeks to characterize the behavioral phenotype of the animals. All behavioral procedures were performed by the same experimenter in the active phase of the animal under dim red light between 7 a.m. and 1 p.m. Animals were habituated to the behavioral rooms 1 h before testing. Additional habituation phases to the arenas were performed before the Novel Object Recognition (duration: 1 h) and Social Interaction (duration: 20 min) test 24 h before testing. All arenas were thoroughly cleaned between each trial using 85% ethanol. Tests were scored by an observer blinded to treatment. All behavioral tests were performed according to established protocols in our facility and all animals underwent the behavioral tests in the following order: Novel Object Recognition Test,^27^ Y‐Maze,^27^ Elevated Plus Maze,^28^ Social Interaction, Pre‐Forced Swim Test, Open Field,^29^ and Forced Swim Test.^29^ The order of the animals within each test was randomized. Social interaction was performed by putting two unfamiliar weight‐matched rats with the same treatment in an Open Field arena for 10 min. The time spent sniffing was regarded as social behavior.

### 2.6 Data Analysis

The primary outcomes of the study were differences in methylation markers (SAM, betaine, choline, and dimethylglycine) between intervention groups. The secondary outcomes were differences in monoamines, liver markers, and behavioral tests. Interventions with the same outcomes were not available to predict the study power, thus the present study is explorative in nature and sample size was based on similar animal intervention studies.^30^, ^31^ From the magnitudes of the differences between FRL and FSL rats shown in **Tables** 1–3 and Supporting Information Table 5, we predicted that an intervention that would revert plasma concentrations of betaine, choline, and dimethylglycine, liver SAM, and urinary choline and dimethylglycine to control levels would require between 7 and 14 rats in each treatment subgroup (*α* = 0.05, power = 80%).

**Table 1:** Plasma and tissue concentrations of C1 metabolites in Flinders Resistant Line (FRL) rats and Flinders Sensitive Line (FSL) rats treated with vehicle^a^ for 10 weeks

	FRL (*n* = 8)	FSL (*n* = 7)	*p* b
Age at the end of the treatment [weeks]	20.5	21.0	0.191
Weight at the end of the treatment [g]	450	393	0.014
Weight increase from baseline [g]	132	116	0.152
EDTA plasma			
Taurine [μmol L^−1^]	140 ± 24.7	162 ± 31.5	0.142
Betaine [μmol L^−1^]	184 ± 17.6	248 ± 45.6	0.001^c^
Choline [μmol L^−1^]	9.04 ± 3.96	13.3 ± 4.43	0.070
Dimethylglycine [μmol L^−1^]	5.61 ± 1.35	7.17 ± 0.84	0.021
Homocysteine [μmol L^−1^]	7.09 ± 0.44	5.48 ± 1.21^d^	0.004
Cystathionine [μmol L^−1^]	0.625 ± 0.113	0.548 ± 0.080^d^	0.179
AST [U L^−1^]	182 ± 50.8	229 ± 60.9	0.128
ALT [U L^−1^]	87.3 ± 39.5	101 ± 21.6	0.433
Liver extract [nmol g^−1^ tissue]			
SAH	59.9 ± 6.63	56.7 ± 5.99	0.340
SAM	68.3 ± 15.0	54.2 ± 10.1	0.055
Hippocampal extract [nmol g^−1^ tissue]			
SAH	1.37 ± 0.25	1.38 ± 0.31	0.942
SAM	28.2 ± 2.67	28.6 ± 2.43	0.738
Prefrontal cortex extract [nmol g^−1^ tissue]			
SAH	1.50 ± 0.18	1.59 ± 0.23	0.425^c^
SAM	24.0 ± 2.16	27.0 ± 2.48	0.026

Data are presented as mean ± SD.

a Vehicle treatment consisted of xylitol, maize‐derived maltodextrin, plum flavor, and malic acid.

b *p*‐Values were determined with the use of a one‐way ANOVA test.

c *p*‐Values were determined with the use of a one‐way ANOVA test performed on log‐transformed data.

d *n* = 6.

ALT, alanine transaminase; AST, aspartate transaminase; SAH, S‐adenosylhomocysteine; SAM, S‐adenosylmethionine.

**Table 2:** Plasma and tissue concentrations of C1 metabolites in Flinders Sensitive Line rats after 10 weeks of vehicle or probiotic intake at two different doses

	Vehicle^a^	109^i^^ ^[CFU d^−1^]^b^	10^10 ^[CFU d^−1^] b	*p* c
	(*n* = 7)	(*n* = 7)	(*n* = 8)	
EDTA plasma				
Taurine [μmol L^−1^]	162 ± 31.5	179 ± 21.2	197 ± 44.5	0.173
Betaine [μmol L^−1^]	248 ± 45.6	233 ± 24.3	205 ± 26.8	0.050^d^, ^e^
Choline [μmol L^−1^]	13.3 ± 4.43	15.8 ± 2.23	15.0 ± 2.53	0.350
Betaine/choline ratio	20.1 ± 6.35	14.9 ± 2.24	14.2 ± 3.55	0.036^f^
Dimethylglycine [μmol L^−1^]	7.17 ± 0.84	7.56 ± 1.88	6.90 ± 1.91	0.743
Homocysteine [μmol L^−1^]	5.48 ± 1.21^g^	7.30 ± 3.56^g^	6.34 ± 1.45^h^	0.407
Cystathionine [μmol L^−1^]	0.548 ± 0.080^g^	0.640 ± 0.128^g^	0.608 ± 0.125^h^	0.383
AST [U L^−1^]	229 ± 60.9	228 ± 33.7	227 ± 52.8	0.997
ALT [U L^−1^]	101 ± 21.6	88.7 ± 18.3	80.1 ± 18.8	0.149
Liver extract [nmol g^−1^ tissue]				
SAH	56.7 ± 5.99	58.4 ± 18.8	64.1 ± 18.4	0.632
SAM	54.2 ± 10.1	52.0 ± 7.72	69.4 ± 11.9	0.006^i^
Hippocampal extract [nmol g^−1^ tissue]				
SAH	1.38 ± 0.31	1.38 ± 0.29	1.37 ± 0.28	0.995
SAM	28.6 ± 2.43	27.3 ± 1.69	26.4 ± 4.35	0.410
Prefrontal cortex extract [nmol g^−1^ tissue]				
SAH	1.59 ± 0.23	1.72 ± 0.53	1.57 ± 0.24	0.834^d^
SAM	27.0 ± 2.48	25.7 ± 1.46	24.7 ± 2.06	0.115

Data are presented as mean ± SD.

a Vehicle treatment consisted of xylitol, maize‐derived maltodextrin, plum flavor, and malic acid.

b Probiotic treatment additionally included *Lactobacillus helveticus* R0052 and *Bifidobacterium longum* R0175 at doses of 10^9^ or 10^10^ CFU d^−1^.

c *p*‐Values were determined with the use of a one‐way ANOVA test followed by Dunnett's post‐hoc test when the ANOVA was significant.

d *p*‐Values were determined with the use of a one‐way ANOVA test performed on log‐transformed data.

e Post‐hoc comparison of vehicle versus 10^10 ^CFU d^−1^ (*p* = 0.042).

f Post‐hoc comparison of vehicle versus 10^10 ^CFU d^−1^ (*p* = 0.030).

g *n* = 6.

h *n* = 7.

i Post‐hoc comparison of vehicle versus 10^10 ^CFU d^−1^ (*p* = 0.017).

ALT, alanine transaminase; AST, aspartate transaminase; CFU, colony‐forming units; SAH, S‐adenosylhomocysteine; SAM, S‐adenosylmethionine.

**Table 3:** Concentrations of C1 metabolites in urine and stool extracts from Flinders Sensitive Line rats after 10 weeks of vehicle or probiotic intake at two different doses

	Vehicle^a^	10^9 ^[CFU d^−1^]^b^	10^10 ^[CFU d^−1^]^b^	*p* c
	(*n* = 7)	(*n* = 7)	(*n* = 8)	
Urine				
Taurine [mmol L^−1^]	4.42 ± 3.13	3.36 ± 1.49	3.57 ± 5.27	0.854
Betaine [μmol L^−1^]	733 ± 1220	253 ± 52.1^d^	195 ± 118^d^	0.381
Choline [μmol L^−1^]	48.1 ± 15.6	49.1 ± 10.1^d^	43.6 ± 21.7^d^	0.829
Dimethylglycine [μmol L^−1^]	178 ± 121	132 ± 38.4^d^	97.6 ± 64.2^d^	0.162^e^
Water stool extract				
Betaine [nmol L^−1^]	<LOD	<LOD	<LOD	–
Choline [nmol L^−1^]	2.37 ± 0.74	3.07 ± 1.78	1.90 ± 0.42	0.149
Dimethylglycine [nmol L^−1^]	<LOD	<LOD	<LOD	–
SAM [nmol g^−1^ stool]	34.5 ± 18.2	35.8 ± 32.7	30.5 ± 10.2	0.886

Data are presented as mean ± SD.

a Vehicle treatment consisted of xylitol, maize‐derived maltodextrin, plum flavor, and malic acid.

b Probiotic treatment additionally included *Lactobacillus helveticus* R0052 and *Bifidobacterium longum* R0175.

c *p*‐Values were determined with the use of a one‐way ANOVA test.

d *n* = 6.

e *p*‐Values were determined with the use of a one‐way ANOVA test performed on log‐transformed data.

CFU, colony‐forming units; SAM, S‐adenosylmethionine.

All statistics were performed using IBM SPSS 24.0 (IBM Corp., Armonk, NY, USA). Assumptions of normality and homogeneity of variances were tested using Shapiro‐Wilk test and Levene's test, respectively, with both strain and intervention included in the factor list. For variables violating normality or homogeneity of variances (*p* < 0.05), log‐transformed variables were created. If these log‐transformed variables were then able to meet the parametric assumptions (i.e., *p* > 0.05 in both Shapiro‐Wilk and Levene's tests), they were kept as log‐transformed variables throughout the statistical analysis; if the assumptions were still violated upon log‐transformation, the variable was left untransformed. The following variables were log‐transformed: plasma betaine, plasma norepinephrine, plasma 5‐HIAA, hippocampal DOPAC, prefrontal cortex SAH, prefrontal cortex DOPAC, prefrontal cortex dopamine, prefrontal cortex 5‐HIAA, and urinary dimethylglycine. Log‐transformation did not affect the overall interpretation of the data, as no *p*‐values changed from a nonsignificant level to a significant/trend level or vice versa. Bodyweight was included as a covariate in preliminary analyses, but was never significant. Therefore, metabolite concentrations were not corrected for bodyweight.

Results are expressed as mean ± standard deviation (SD). Two separate one‐way analysis of variance (ANOVA) tests were used to study differences in concentrations of the biomarkers between FSL and FRL rats and between the three intervention groups. A significant ANOVA was followed by post‐hoc pairwise comparison of probiotic treatment with vehicle using Dunnett's procedure to correct for multiple comparisons. *p*‐Values < 0.05 (two‐tailed) were considered statistically significant and *p*‐values between 0.05 and 0.10 were considered to indicate a tendency.

## 3 Results

Supporting Information Figure 2 shows the growth curve of the rats according to the strain and the intervention type. In FSL rats, weight and weight gain (from baseline to week 10 of the intervention) did not differ between intervention groups at any time point. There were no significant differences in water or food intake between the two strains or the three intervention groups (data not shown).

### 3.1 Strain Effects on C1‐Related Metabolites in FSL Versus FRL Rats

FSL and FRL rats showed no differences in plasma concentrations of cystathionine (Table 1). Compared with FRL rats, FSL rats showed higher plasma concentrations of betaine (mean = 248 vs. 184 μmol L^−1^; *F*(1,13) = 16.93, *p* = 0.001) and dimethylglycine (7.17 vs. 5.61 μmol L^−1^; *F*(1,13) = 6.92, *p* = 0.021). Concentrations of plasma choline tended to be higher in FSL than FRL rats (13.3 vs. 9.04 μmol L^−1^; *F*(1,13) = 3.90, *p* = 0.070). FSL rats had lower concentrations of plasma homocysteine (5.48 vs. 7.09 μmol L^−1^; *F*(1,12) = 12.17, *p* = 0.004).

Concentrations of SAH in liver tissue extracts did not differ significantly between the two strains, whereas SAM in liver extracts tended to be lower in FSL than in FRL rats (54.2 vs. 68.3 nmol g^−1^ tissue; *F*(1,13) = 4.44, *p* = 0.055). SAM concentrations in hippocampal extracts were approximately 2‐fold lower than in liver extracts, but did not differ between the strains. Similarly, hippocampal SAH was unchanged between FSL and FRL rats. In the prefrontal cortex, FSL rats had higher concentrations of SAM than FRLs (27.0 vs. 24.0 nmol g^−1^ tissue; *F*(1,13) = 6.32, *p* = 0.026), but the strains did not differ in SAH or SAM/SAH ratio.

The urinary excretion of betaine did not differ significantly between the strains. FSL rats showed 56% lower urinary excretion of choline (48.1 vs. 110 μmol L^−1^; *F*(1,13) = 14.53, *p* = 0.002) and 200% higher urinary excretion of dimethylglycine (178 vs. 57.0 μmol L^−1^; *F*(1,13) = 11.84, *p* = 0.004) than FRL rats (Supporting Information Table 4). The concentrations of betaine, choline, and dimethylglycine in stool extracts were very low, suggesting that the dietary nutrients, choline and betaine, had been utilized and dimethylglycine was not present or produced by the gut bacteria in a significant amount. The concentrations of SAM in stool extracts were remarkably high (in mmol L^−1^ range) but did not differ between the strains (Supporting Information Table 4). In contrast, the molar concentrations of SAH in stool extracts were very low compared with SAM, which prevented the analysis of SAH and SAM in the same run (as it was the case for all other biological matrices).

### 3.2 Intervention Effects on C1‐Related Metabolites in FSL Rats

Table 2 shows the intervention‐induced changes in concentrations of the C1‐related metabolites measured in different biological samples from FSL rats. Compared with FSL rats receiving vehicle, FSL rats treated with high‐dose probiotics had lower plasma concentrations of betaine (248 vs. 205 μmol L^−1^; main effect ANOVA: *F*(2,19) = 3.54, *p* = 0.050; post‐hoc: *p* = 0.042) and a lower betaine/choline ratio (20.1 vs. 14.2; ANOVA: *F*(2,19) = 4.00, *p* = 0.036; post‐hoc: *p* = 0.030). Concentrations of SAM in liver extracts were significantly higher in FSL rats treated with high‐dose probiotics as compared with vehicle (69.4 vs. 54.2 nmol g^−1^ tissue; ANOVA: *F*(2,19) = 6.66, *p* = 0.006; post‐hoc: *p* = 0.017). Concentrations of SAM in hippocampal or prefrontal cortex samples did not differ significantly between intervention groups. Urinary and stool excretions of the metabolites did not show significant differences between the three interventions (Table 3).

### 3.3 Strain Effects on Monoamine Metabolites in FSL Versus FRL Rats

Plasma concentrations of norepinephrine tended to be higher in FSL than FRL rats (40.9 vs. 29.1 nmol L^−1^; *F*(1,13) = 4.41, *p* = 0.056) (**Table** 4). FSL rats also had higher plasma concentrations of DOPAC (15.3 vs. 10.1 nmol L^−1^; *F*(1,13) = 14.77, *p* = 0.002), dopamine (19.2 vs. 12.9 nmol L^−1^; *F*(1,13) = 15.81, *p* = 0.002), and 5‐HT (1174 vs. 311 nmol L^−1^; *F*(1,13) = 8.51, *p* = 0.012). The strains did not differ in plasma 5‐HIAA. Plasma DOPAC/dopamine and 5‐HIAA/5‐HT ratios, commonly used as indicators for dopamine and serotonin turnover, respectively, did not differ between the strains.

**Table 4:** Plasma and tissue concentrations of monoamine neurotransmitters in FRL and FSL rats treated with vehicle^a^ for 10 weeks

	FRL (*n* = 8)	FSL (*n* = 7)	*p* b
EDTA plasma [nmol L^−1^]			
Norepinephrine	29.1 ± 11.1	40.9 ± 13.3	0.056^c^
Dopamine	12.9 ± 3.13	19.2 ± 2.94	0.002
DOPAC	10.1 ± 1.48	15.3 ± 3.52	0.002
5‐HT	311 ± 248	1174 ± 798	0.012
5‐HIAA	18.3 ± 2.78	19.1 ± 8.36	0.894^c^
Hippocampal extract [nmol g^−1^ tissue]			
Norepinephrine	1.25 ± 0.16	1.40 ± 0.13	0.075
Dopamine	0.02 ± 0.01	0.02 ± 0.004	0.596
DOPAC	0.01 ± 0.004	0.01 ± 0.003	0.453^c^
5‐HT	0.37 ± 0.10	0.40 ± 0.06	0.443
5‐HIAA	0.36 ± 0.03	0.39 ± 0.14	0.504
Prefrontal cortex extract [nmol g^−1^ tissue]			
Norepinephrine	0.66 ± 0.09	0.91 ± 0.14	0.001
Dopamine	0.43 ± 0.45	0.66 ± 0.61	0.520^c^
DOPAC	0.45 ± 0.44	0.37 ± 0.31	0.657^c^
5‐HT	0.28 ± 0.07	0.42 ± 0.08	0.004
5‐HIAA	0.39 ± 0.07	0.42 ± 0.15	0.635^c^

Data are presented as mean ± SD.

a Vehicle treatment consisted of xylitol, maize‐derived maltodextrin, plum flavor, and malic acid.

b *p*‐Values were determined with the use of a one‐way ANOVA test.

c *p*‐Values were determined with the use of a one‐way ANOVA test performed on log‐transformed data.

5‐HIAA, 5‐hydroxyindoleacetic acid; 5‐HT, 5‐hydroxytryptamine (serotonin); DOPAC, 3,4‐dihydroxyphenylacetic acid.

FSL rats tended to have higher concentrations of norepinephrine in the hippocampus than FRLs (1.40 vs. 1.25 nmol g^−1^ tissue; *F*(1,13) = 3.73, *p* = 0.075), but they did not differ in hippocampal DOPAC, dopamine, 5‐HT, or 5‐HIAA (Table 4). In accordance with plasma and hippocampal results, FSL rats also had higher concentrations of norepinephrine in the prefrontal cortex than FRL rats (0.91 vs. 0.66 nmol g^−1^ tissue; *F*(1,13) = 18.10, *p* = 0.001) (Table 4). While DOPAC and dopamine levels in prefrontal cortex did not differ between the strains, FSL rats had lower dopamine turnover (i.e., DOPAC/dopamine ratio) in the prefrontal cortex compared to FRL rats (0.62 vs. 1.08; *F*(1,13) = 12.17, p < 0.001). 5‐HT concentrations in the prefrontal cortex were higher in FSLs compared to FRLs (0.42 vs. 0.28 nmol g^−1^ tissue; *F*(1,13) = 12.55, *p* = 0.004). There were no differences in 5‐HIAA or serotonin turnover.

### 3.4 Intervention Effects on Monoamine Metabolites in FSL Rats

Differences in monoamine concentrations according to the intervention are shown in **Table** 5. FSLs treated with low‐dose probiotics had lower plasma concentrations of norepinephrine than vehicle‐treated ones (27.2 vs. 40.9 nmol L^−1^; ANOVA: *F*(2,19) = 5.30, *p* = 0.015; post‐hoc: *p* = 0.033). Compared to vehicle, FSLs receiving low‐dose probiotics had lower plasma concentrations of dopamine (19.2 vs. 14.5 nmol L^−1^; ANOVA: *F*(2,19) = 6.73, *p* = 0.006; post‐hoc: *p* = 0.027. Compared to vehicle, FSLs receiving high‐dose probiotics also had lower plasma concentrations of dopamine (19.2 vs. 13.2 nmol L^−1^; *p* = 0.004). The DOPAC/dopamine ratio was higher in the high‐dose probiotic group than in the vehicle group (1.35 vs. 0.81; ANOVA: *F*(2,19) = 3.73, *p* = 0.043; post‐hoc: *p* = 0.036). There were no significant differences in hippocampal or prefrontal cortex neurotransmitters between intervention groups.

**Table 5:** Plasma and tissue concentrations of monoamine neurotransmitters in Flinders Sensitive Line (FSL) rats after 10 weeks of vehicle^a^ or probiotic^b^ intake at two different doses

	Vehicle	10^9 ^[CFU d^−1^]	10^10 ^[CFU d^−1^]	*p* c
	(*n* = 7)	(*n* = 7)	(*n* = 8)	
EDTA plasma; nmol L^−1^				
Norepinephrine	40.9 ± 13.3	27.2 ± 6.60	42.7 ± 12.1	0.015^d^^,^ e
Dopamine	19.2 ± 2.94	14.5 ± 2.61	13.2 ± 4.01	0.006^f^
DOPAC	15.3 ± 3.52	13.1 ± 3.18	16.5 ± 5.49	0.306
5‐HT	1174 ± 789	1240 ± 712	1329 ± 699	0.920
5‐HIAA	19.1 ± 8.36	16.1 ± 4.30	15.1 ± 2.50	0.494^d^
Hippocampal extract [nmol g^−1^ tissue]				
Norepinephrine	1.40 ± 0.13	1.51 ± 0.20	1.33 ± 0.26	0.266
Dopamine	0.02 ± 0.004	0.03 ± 0.01	0.02 ± 0.01	0.244
DOPAC	0.01 ± 0.003	0.01 ± 0.01	0.01 ± 0.001	0.243^d^
5‐HT	0.40 ± 0.06	0.43 ± 0.10	0.40 ± 0.09	0.814
5‐HIAA	0.39 ± 0.14	0.34 ± 0.07	0.33 ± 0.09	0.515
Prefrontal cortex extract [nmol g^−1^ tissue]				
Norepinephrine	0.91 ± 0.14	0.88 ± 0.09	0.84 ± 0.13	0.620
Dopamine	0.66 ± 0.61	0.27 ± 0.18	0.49 ± 0.41	0.383^d^
DOPAC	0.37 ± 0.31	0.19 ± 0.10	0.31 ± 0.29	0.601^d^
5‐HT	0.42 ± 0.08	0.36 ± 0.06	0.38 ± 0.13	0.511
5‐HIAA	0.42 ± 0.15	0.37 ± 0.06	0.34 ± 0.08	0.296^d^

Data are presented as mean ± SD.

a Vehicle treatment consisted of xylitol, maize‐derived maltodextrin, plum flavor, and malic acid.

b Probiotic treatment additionally included *Lactobacillus helveticus* R0052 and *Bifidobacterium longum* R0175.

c *p*‐Values were determined with the use of a one‐way ANOVA test followed by Dunnett's post‐hoc test when the ANOVA was significant.

d *p*‐Values were determined with the use of a one‐way ANOVA test performed on log‐transformed data.

e Post‐hoc comparison of vehicle versus 10^9 ^CFU d^−1^ (*p* = 0.033).

f Post‐hoc comparison of vehicle versus 10^9 ^CFU d^−1^ (*p* = 0.027), vehicle versus 10^10 ^CFU d^−1^ (*p* = 0.004).

5‐HIAA, 5‐hydroxyindoleacetic acid; 5‐HT, 5‐hydroxytryptamine (serotonin); DOPAC, 3,4‐dihydroxyphenylacetic acid.

Supporting Information Table 7 provides the results of a stepwise multiple linear regression analysis to predict concentrations of monoamines (dependent variables) in FSL rats. Remarkably, 84% of the variability in plasma dopamine between FSL rats was explained by a model containing the following independent variables: treatment, plasma dimethylglycine, cystathionine, taurine, and hippocampal SAM. Different C1 metabolites were significant determinants for plasma serotonin, 5‐HIAA, and prefrontal cortex dopamine.

### 3.5 Liver Function Markers

Concentrations of plasma ALT and AST did not differ between strains (Table 1) or intervention groups (Table 2). The AST/ALT ratio was higher in the high‐dose probiotic group compared to vehicle (2.89 vs. 2.25; ANOVA: *F*(2,19) = 3.77, *p* = 0.042; post‐hoc: *p* = 0.024).

### 3.6 Behavioral Tests

Vehicle‐treated FSL rats moved a greater distance in the Open Field than FRL rats (3783 vs. 2884 cm; *F*(1,13) = 9.40, *p* = 0.009). Moreover, FSL rats were more immobile (144 vs. 101 s; *F*(1,13) = 13.50, *p* = 0.003) and swam less (56.4 s vs. 93.8 s; *F*(1,13) = 10.13, *p* = 0.007) in the pre‐Forced Swim Test than FRL rats. The same differences in immobility and swimming behavior were observed in the test session 24 h later (Supporting Information Table 5). These results have been reported previously and confirm the validity of the depression model. FSL and FRL rats did not differ in nonspatial memory, spatial memory, anxiety, or social behavior. There were no significant differences between the intervention groups.

## 4 Discussion

Our study provided novel evidence for probiotics influencing two main metabolic pathways in the host. First, depressed rats had lower liver concentrations of the methyl donor SAM than control rats, which was increased by administration of *L. helveticus* R0052 and *B. longum* R0175 (10^10 ^CFU d^−1^). Second, plasma dopamine was elevated in the depressed compared with the control rats, which was lowered by probiotics in a dose‐dependent manner without changes in the dopamine catabolite DOPAC. The influence of probiotics on two interrelated biochemical pathways involved in mood disorders (i.e., C1 and catecholamine metabolisms) could be driven by interrelated or independent mechanisms as discussed below.

### 4.1 Effects of Probiotics on C1 Metabolism

Higher plasma concentrations of betaine, choline, and dimethylglycine in addition to lower plasma homocysteine in vehicle‐treated FSL rats compared with FRL rats suggest enhanced homocysteine remethylation and SAM production via the betaine‐homocysteine methyltransferase (BHMT) pathway (Supporting Information Figure 3). Treatment with probiotics (10^10 ^CFU d^−1^) reduced the metabolic dependency of the rats on betaine and choline as methyl donors, and increased liver SAM to levels comparable with control animals. Vehicle‐treated FSL rats had higher SAM concentrations in the prefrontal cortex than FRL rats, potentially indicating higher requirements for SAM. Probiotics did not affect SAM in the brain but increased SAM in the host's liver. The liver is the main SAM‐producing organ, but it remains unclear whether the increase in liver SAM is due to the rats’ own synthesis.

Lactic acid bacteria in fermented Cheonggukjang have been shown to produce SAM in vitro,^9^, ^32^ suggesting that SAM produced through gut bacterial fermentation could be available to the host. Alterations in host C1 metabolism in the probiotic group could be due to the bacteria's own production of SAM (GenomeNet Database Resources^33^) (Supporting Information Table 6). The SAM amount in stool samples (i.e., second‐highest after the liver: mean approximately 34 nmol g^−1^ stool wet weight) suggests that bacteria‐driven fermentation processes in the gut could constitute a significant, yet underestimated source of SAM for the host (Supporting Information Figure 3). Alternatively, lowered methyl group flow through the BHMT pathway and increased liver SAM in animals treated with high‐dose probiotics could be mediated by saving tetrahydrobiopterin (BH4) and SAM due to lowering dopamine as discussed below.

### 4.2 Effects of Probiotics on Monoamines

Compared to FRL rats, FSLs had higher plasma and brain (hippocampus and prefrontal cortex) concentrations of norepinephrine. Dopamine, norepinephrine's precursor, was increased only in plasma. This distinct increase has been described previously,^34^ and has been suggested to reflect the depressive‐like phenotype of FSL rats.

Compared to vehicle, probiotics were associated with a dose‐dependent decline in plasma dopamine and a reduction in plasma norepinephrine (only in the low‐dose group). Previous studies reported heterogeneous effects of probiotics on catecholamines, likely due to using different animal models, tissues, and bacterial strains. *Bifidobacterium infantis* reversed the stress‐induced decrease of norepinephrine (i.e., increased norepinephrine) in the brainstem of Sprague‐Dawley rats.^35^ *L. helveticus* R0052 and *B. longum* R0175 (10^9 ^CFU d^−1^, corresponding to our low dose) attenuated the stress‐induced increase of plasma norepinephrine (i.e., lowered norepinephrine) in mice.^36^ In our study, dopamine and norepinephrine were only lowered in plasma after probiotic treatment but not in the brain, suggesting that the extraneuronal pathways of metabolism and/or uptake of these catecholamines were modified by the intervention.

**Figure 1:**
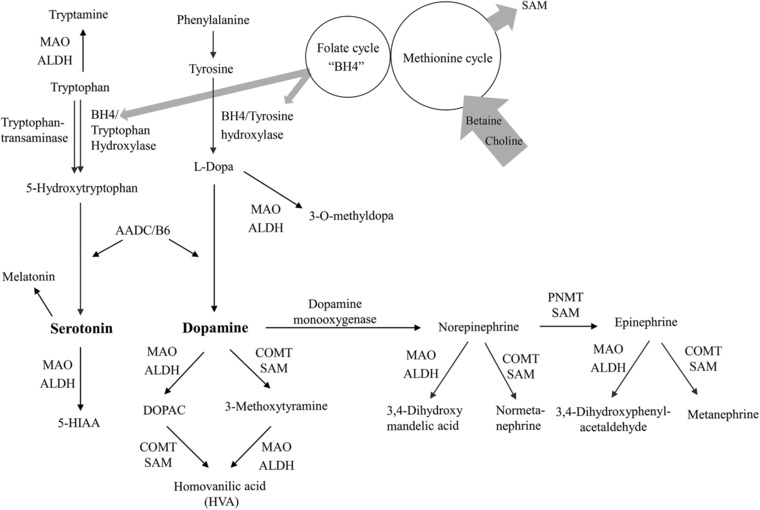
Metabolism of catecholamines and its interactions with C1 metabolism via S‐adenosylmethionine (SAM) and tetrahydrobiopterin (BH4). 5‐HIAA, 5‐hydroxyindole acetic acid; 5‐HT, 5‐hydroxytryptamine (serotonin); AADC, aromatic l‐amino acid decarboxylase; ALDH, aldehyde dehydrogenase; COMT, catechol‐O‐methyltransferase; DOPAC, 3,4‐dihydroxyphenylacetic acid; MAO, monoaminoxydase; PNMT, phenylethanolamine *N*‐methyltransferase.

Extraneuronal sites of catecholamine synthesis (adrenal medulla for norepinephrine; gastrointestinal tract and kidney for dopamine) could represent target organs for probiotics. Lowering of plasma dopamine could be linked to enhanced gastrointestinal motility.^37^ Previous intervention studies with probiotics in mice showed no changes in levels of serotonin and dopamine in the colon and the small intestine, although behavioral changes were detected.^38^ The decline in plasma dopamine in our study may be theoretically related to alterations in its extraneuronal uptake, less synthesis from l‐dopa, or enhanced degradation via COMT (SAM dependent) or via monooxygenase (MAO) to norepinephrine (**Figure** 1). However, because DOPAC was not different, and norepinephrine was not different or even lower in probiotic‐treated animals, it is more likely that less dopamine was formed or more dopamine was excreted instead of catabolized. The main sources of plasma dopamine are the sympathetic nerves, the adrenal medulla, and the gastrointestinal tract,^39^ while the proportional contribution of brain dopamine to plasma dopamine is minor, as dopamine cannot cross the blood–brain barrier.^39^ Plasma dopamine is thought to be mainly excreted in urine and only partly converted to noradrenaline.^39^

Low plasma dopamine, associated with lower plasma norepinephrine in the low‐dose probiotics group only, was unlikely due to enhanced metabolism to DOPAC or norepinephrine (via MAO). Instead, dopamine lowering in the probiotic groups is likely due to less dopamine synthesis from l‐dopa, tyrosine, phenylalanine, or more dopamine excreted in urine. The effects on plasma dopamine were stronger than on norepinephrine and showed dose‐dependency, suggesting that dopamine is likely to be closer to an upstream pathway (i.e., l‐dopa) where probiotics would interfere with catecholamine metabolism.

Elevated serotonin in plasma and prefrontal cortex of FSL has been reported before.^40^ The intervention did not affect serotonin or its catabolite 5‐HIAA. FSL rats appear to have higher production of serotonin from tryptophan and dopamine from phenylalanine; both reactions are catalyzed by intracellular aromatic l‐amino acid decarboxylase (AADC/B6 dependent). Inhibition of AADC by probiotics is very unlikely, because probiotics specifically lowered dopamine but not serotonin.

### 4.3 Interrelations Between C1 and Catecholamine Metabolisms

Elevated dopamine and serotonin in FSL rats could challenge C1 metabolism in at least two ways. High dopamine input may deplete SAM (i.e., COMT and PNMT are SAM‐dependent). Moreover, production of serotonin from tryptophan (via tryptophan hydroxylase) and dopamine from phenylalanine (via tyrosine and tyrosine hydroxylase) requires tetrahydrobiopterin (BH4) (Figure 1). This could deplete C1 units of folate and place more pressure on BHMT to provide SAM from betaine. Patients with depression are known to have low BH4^41^ and folate.^42^ Moreover, elevated serotonin has been reported to bind to COMT and overlap with SAM‐binding sites, thereby inhibiting COMT by preventing SAM binding.^43^ Therefore, high serotonin in vehicle‐treated FSL rats could have caused reduced dopamine degradation via SAM‐dependent COMT. *Lactobacillus* strains produce SAM in vitro^9^, ^32^ and *L. helveticus* is also able to produce C1 units and metabolize phenylalanine and tyrosine via transamination and dehydrogenation pathways,^44^ suggesting that they could have provided BH4 or other intermediate metabolites of phenylalanine and tyrosine that could have influenced dopamine production in extraneuronal tissues.

### 4.4 Effects of Probiotics on Liver Function Markers and Behavior

There were no statistically significant effects of probiotics on liver function markers (AST, ALT) or behavior (cognition, anxiety, locomotion, depression). The literature is not consistent about effects of probiotics on these health outcomes. A slight decrease in plasma ALT, along with increased liver SAM after probiotic treatment, may be indicative of protective effects of probiotics on the liver. Probiotics did not significantly affect behavior in the Forced Swim Test, although mean immobility values were slightly lower in the probiotic group. The lack of changes in behavioral tests is consistent with unchanged monoamine neurotransmitters in the brain, as these outcomes often coincide.

### 4.5 Limitations and Conclusions

The limitations of the current study deserve mentioning. First, the relatively low sample size may have limited the study power to detect some differences. Furthermore, such differences may have been attenuated by the methyl donors present in the standard diet. However, since the metabolites were independently measured in several biological matrices, and the independent comparisons of the strain and the intervention effects supported the hypothesis, it is very unlikely that the results were due to chance.

Taken together, probiotic intervention was associated with higher liver SAM and attenuated metabolic flow of methyl groups from betaine to dimethylglycine via BHMT. In addition, probiotics lowered plasma concentrations of dopamine and norepinephrine, but did not influence brain monoamines. Future studies need to investigate whether the increase of SAM was due to bacterial metabolism by using the methyl donors available in the host diet. Future studies may investigate whether administration of probiotics together with selected nutrients may serve as supportive treatment for C1 metabolism–related diseases. Furthermore, the health impacts of lowering extraneuronal dopamine and norepinephrine by probiotics deserve further investigations. At this early stage, no direct extrapolation to humans can be made.

**Abbreviations**
5‐HIAA: 5‐hydroxyindoleacetic acid
AADC: aromatic l‐amino acid decarboxylase
ALT: alanine transaminase
AST: aspartate transaminase
BH4: tetrahydrobiopterin
BHMT: betaine‐homocysteine methyltransferase
CFU: colony‐forming units
COMT: catechol‐O‐methyltransferase
DOPAC: 3,4‐dihydroxyphenylacetic acid
FRL: Flinders Resistant Line
FSL: Flinders Sensitive Line
MAO: monoaminoxidase
PNMT: phenylethanolamine *N*‐methyltransferase
SAH: S‐adenosylhomocysteine
SAM: S‐adenosylmethionine

## Conflict of Interest

The authors declare no conflict of interest.

## Supporting information


**Supporting Information Figure 1**. Experimental design. Animals were allowed to acclimatize for 2 weeks (week −2 to week 0) before the start of the intervention (probiotics/vehicle) from week 0 to week 10. Behavioral tests were conducted from week 6 to week 9. After 10 weeks of intervention, animals were euthanized and tissue was collected. CFU, colony‐forming units; FRL, Flinders Resistant Line; FSL, Flinders Sensitive Line. * Vehicle treatment consisted of xylitol, maize‐derived maltodextrin, plum flavor, and malic acid; low dose = 10^9^ CFU d^−1^; high dose = 10^10^ CFU d^−1^.
**Supplemental Figure 2**. Weekly growth curves of FSL and FRL rats assigned to intervention groups from baseline to week 10 and weight increase between baseline and week 10. Vehicle‐treated FSL rats weighed significantly less than FRL rats at baseline (*p* = 0.030). The weight differences were maintained at all weeks until the end of the study. The increase in weight between baseline and week 10 did not differ significantly between vehicle‐treated FSL and FRL rats (*p* = 0.187), or among FSL rats between the treatment arms (*p* = 0.793). Vehicle treatment consisted of xylitol, maize‐derived maltodextrin, plum flavor, and malic acid; low dose = 10^9^ CFU/d; high dose = 10^10^ CFU/d.
**Supplemental Figure 3**. Summary of the proposed effect of probiotics on host C1‐metabolism. The liver is the main SAM‐synthesizing organ. Probiotics that are able to use and/or synthesize methyl donors might be a source of SAM that could support the host's needs. 5‐MTHF, 5‐methyltetrahydrofolate; BHMT, betaine‐homocysteine methyl transferase; SAM, S‐adenosylmethionine; SAH, S‐adenosylhomocysteine; THF, tetrahydrofolate.

SUPPLEMENTAL TABLE **1**. Main nutritional values and composition of nutrients directly related to one‐carbon metabolism in the standard diet (Altromin 1324)
SUPPLEMENTAL TABLE 2. Biosamples collected from the rats; storage and extraction conditions and markers measured in each tissue matrix.
SUPPLEMENTAL TABLE 3. Performance of the methods used in the present paper according to the matrix used for method validation.
SUPPLEMENTAL TABLE 4. Concentrations of C1‐metabolites and related compounds excreted in urine and stool extracts from Flinders Resistant Line (FRL) rats and Flinders Sensitive Line (FSL) rats treated with vehicle^1^ for 10 weeks.
SUPPLEMENTAL TABLE 5. Results of mood‐related behavioural tests performed after 6–9 weeks of intervention with probiotics or vehicle.
SUPPLEMENTAL TABLE 6. Genes coding for enzymes related to C1‐metabolism are present in *Lactobacillus helveticus* R0052 genome.
SUPPLEMENTAL TABLE 7. Stepwise multiple linear regression analysis applied to find predictors of plasma and prefrontal cortex
