# Supplementary material for: Probiotics Affect One‐Carbon Metabolites and Catecholamines in a Genetic Rat Model of Depression
Source: Mol Nutr Food Res. 2018 Mar 13;62(7):1701070. doi: 10.1002/mnfr.201701070 (PMC5900923; doi:10.1002/mnfr.201701070)
Supplement: Supplementary file 1 — Supporting Information Figure 1. Experimental design. Animals were allowed to acclimatize for 2 weeks (week −2 to week 0) before the start of the intervention (probiotics/vehicle) from week 0 to week 10. Behavioral tests were conducted from week 6 to week 9. After 10 weeks of intervention, animals were euthanized and tissue was collected. CFU, colony‐forming units; FRL, Flinders Resistant Line; FSL, Flinders Sensitive Line. * Vehicle treatment consisted of xylitol, maize‐derived maltodextrin, plum flavor, and malic acid; low dose = 109 CFU d−1; high dose = 1010 CFU d−1. Supplemental Figure 2. Weekly growth curves of FSL and FRL rats assigned to intervention groups from baseline to week 10 and weight increase between baseline and week 10. Vehicle‐treated FSL rats weighed significantly less than FRL rats at baseline (p = 0.030). The weight differences were maintained at all weeks until the end of the study. The increase in weight between baseline and week 10 did not differ significantly between vehicle‐treated FSL and FRL rats (p = 0.187), or among FSL rats between the treatment arms (p = 0.793). Vehicle treatment consisted of xylitol, maize‐derived maltodextrin, plum flavor, and malic acid; low dose = 109 CFU/d; high dose = 1010 CFU/d. Supplemental Figure 3. Summary of the proposed effect of probiotics on host C1‐metabolism. The liver is the main SAM‐synthesizing organ. Probiotics that are able to use and/or synthesize methyl donors might be a source of SAM that could support the host's needs. 5‐MTHF, 5‐methyltetrahydrofolate; BHMT, betaine‐homocysteine methyl transferase; SAM, S‐adenosylmethionine; SAH, S‐adenosylhomocysteine; THF, tetrahydrofolate. [file MNFR-62-na-s001.pptx]

## Slide 1
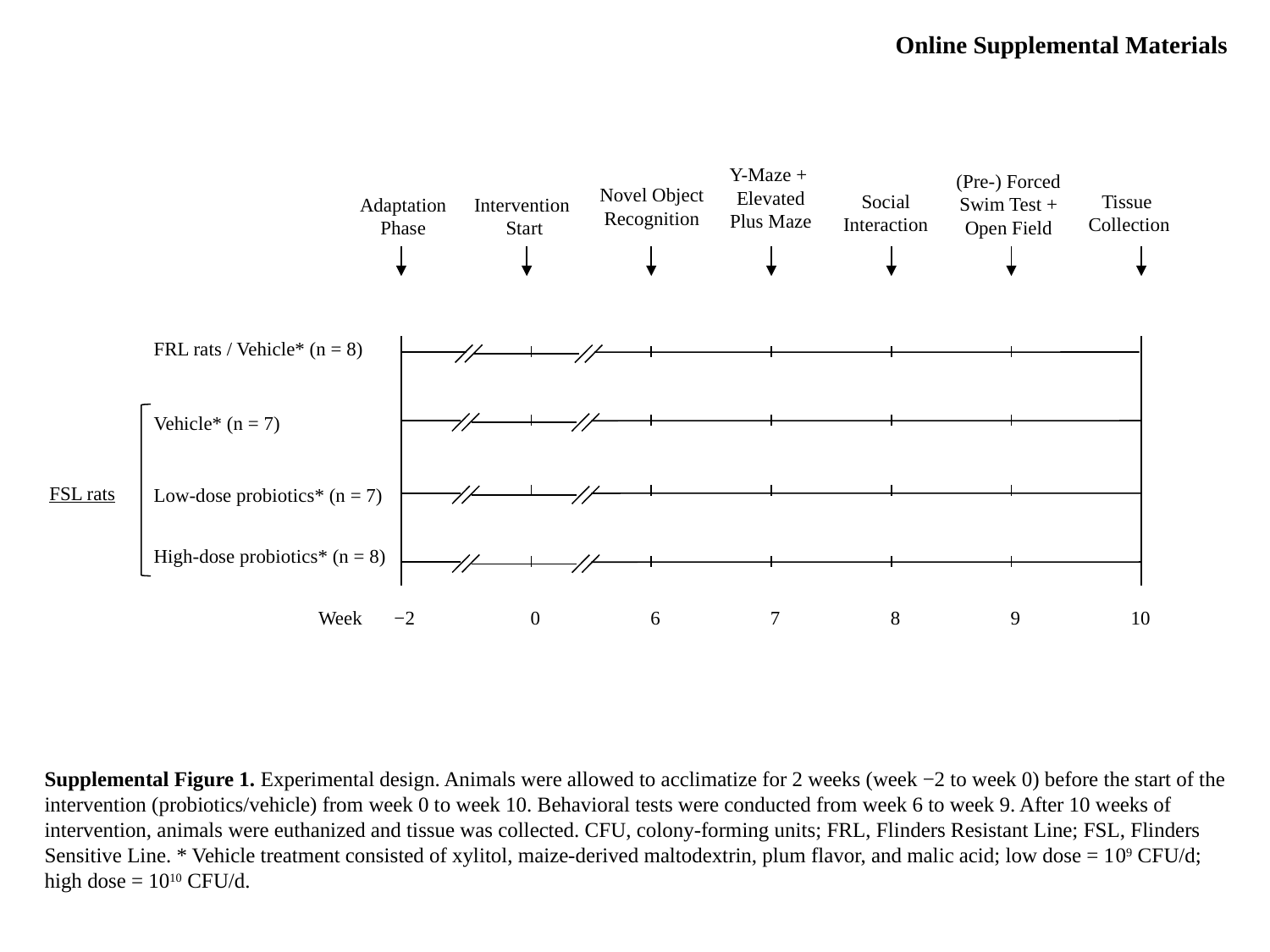

Online Supplemental Materials
Y-Maze +
Elevated Plus Maze
(Pre-) Forced Swim Test + Open Field
Novel Object Recognition
Social Interaction
Tissue
Collection
Intervention
Start
Adaptation Phase
FRL rats / Vehicle* (n = 8)
Vehicle* (n = 7)
FSL rats
Low-dose probiotics* (n = 7)
High-dose probiotics* (n = 8)
10
0
6
7
8
−2
Week
9
Supplemental Figure 1. Experimental design. Animals were allowed to acclimatize for 2 weeks (week −2 to week 0) before the start of the intervention (probiotics/vehicle) from week 0 to week 10. Behavioral tests were conducted from week 6 to week 9. After 10 weeks of intervention, animals were euthanized and tissue was collected. CFU, colony-forming units; FRL, Flinders Resistant Line; FSL, Flinders Sensitive Line. * Vehicle treatment consisted of xylitol, maize-derived maltodextrin, plum flavor, and malic acid; low dose = 109 CFU/d; high dose = 1010 CFU/d.

## Slide 2
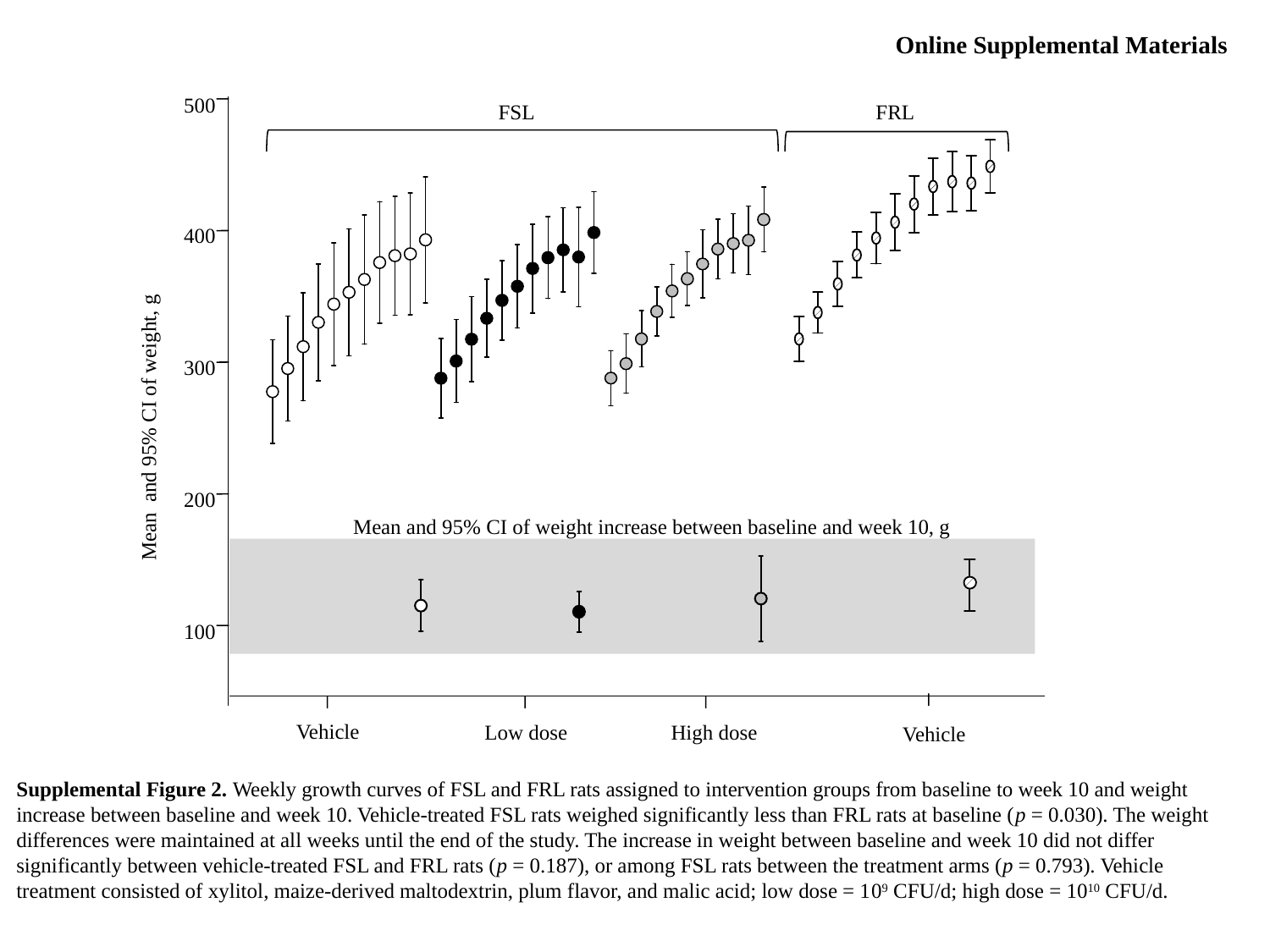

Online Supplemental Materials
500
FRL
FSL
400
300
Mean and 95% CI of weight, g
200
Mean and 95% CI of weight increase between baseline and week 10, g
100
Vehicle
Low dose
High dose
Vehicle
Supplemental Figure 2. Weekly growth curves of FSL and FRL rats assigned to intervention groups from baseline to week 10 and weight increase between baseline and week 10. Vehicle-treated FSL rats weighed significantly less than FRL rats at baseline (p = 0.030). The weight differences were maintained at all weeks until the end of the study. The increase in weight between baseline and week 10 did not differ significantly between vehicle-treated FSL and FRL rats (p = 0.187), or among FSL rats between the treatment arms (p = 0.793). Vehicle treatment consisted of xylitol, maize-derived maltodextrin, plum flavor, and malic acid; low dose = 109 CFU/d; high dose = 1010 CFU/d.

## Slide 3
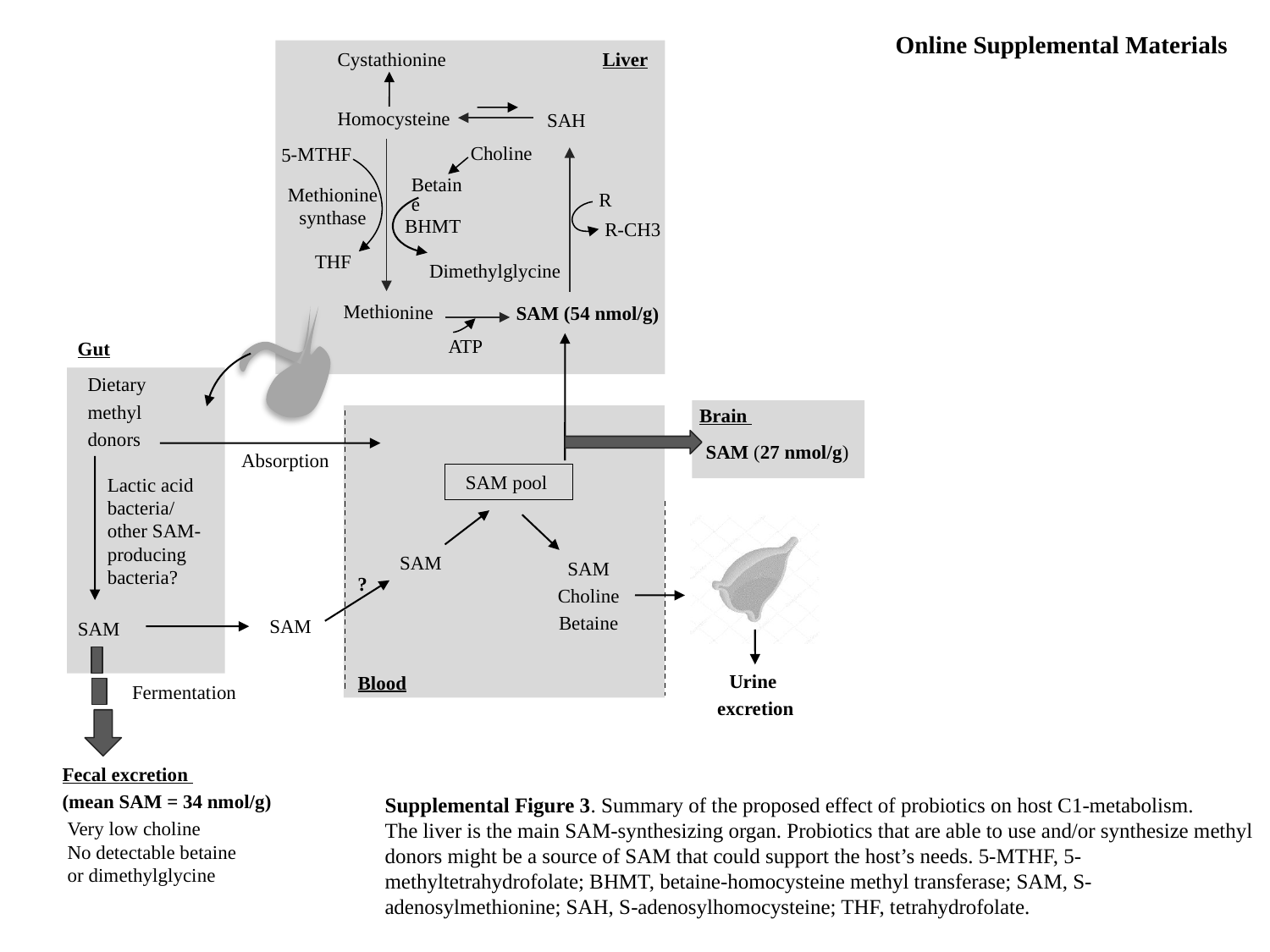

Online Supplemental Materials
Cystathionine
Liver
Homocysteine
SAH
5-MTHF
Choline
Betaine
Methionine synthase
R
BHMT
R-CH3
THF
Dimethylglycine
Methionine
SAM (54 nmol/g)
Gut
ATP
Dietary
methyl
donors
Brain
SAM (27 nmol/g)
Absorption
SAM pool
Lactic acid bacteria/
other SAM-producing bacteria?
SAM
SAM
Choline
Betaine
?
SAM
SAM
Urine
excretion
Blood
Fermentation
Fecal excretion
(mean SAM = 34 nmol/g)
Very low choline
No detectable betaine or dimethylglycine
Supplemental Figure 3. Summary of the proposed effect of probiotics on host C1-metabolism.
The liver is the main SAM-synthesizing organ. Probiotics that are able to use and/or synthesize methyl donors might be a source of SAM that could support the host’s needs. 5-MTHF, 5-methyltetrahydrofolate; BHMT, betaine-homocysteine methyl transferase; SAM, S-adenosylmethionine; SAH, S-adenosylhomocysteine; THF, tetrahydrofolate.
