# Supplementary material for: Probiotics Affect One‐Carbon Metabolites and Catecholamines in a Genetic Rat Model of Depression
Source: Mol Nutr Food Res. 2018 Mar 13;62(7):1701070. doi: 10.1002/mnfr.201701070 (PMC5900923; doi:10.1002/mnfr.201701070)
Supplement: Supplementary file 2 — SUPPLEMENTAL TABLE 1. Main nutritional values and composition of nutrients directly related to one‐carbon metabolism in the standard diet (Altromin 1324) SUPPLEMENTAL TABLE 2. Biosamples collected from the rats; storage and extraction conditions and markers measured in each tissue matrix. SUPPLEMENTAL TABLE 3. Performance of the methods used in the present paper according to the matrix used for method validation. SUPPLEMENTAL TABLE 4. Concentrations of C1‐metabolites and related compounds excreted in urine and stool extracts from Flinders Resistant Line (FRL) rats and Flinders Sensitive Line (FSL) rats treated with vehicle1 for 10 weeks. SUPPLEMENTAL TABLE 5. Results of mood‐related behavioural tests performed after 6–9 weeks of intervention with probiotics or vehicle. SUPPLEMENTAL TABLE 6. Genes coding for enzymes related to C1‐metabolism are present in Lactobacillus helveticus R0052 genome. SUPPLEMENTAL TABLE 7. Stepwise multiple linear regression analysis applied to find predictors of plasma and prefrontal cortex [file MNFR-62-na-s002.docx]

| SUPPLEMENTAL TABLE 1. Main nutritional values and composition of nutrients directly related to one-carbon metabolism in the standard diet (Altromin 1324) | |
| --- | --- |
| Metab. Energy, kcal/kg | 3188 |
| Crude protein, mg/kg | 191970 |
| Crude fat, mg/kg | 40803 |
| Crude fiber, mg/kg | 60518 |
| Methionine, g/kg | 2.7 |
| Choline chloride, mg/kg | 699 |
| Vitamin B12, mg/kg | 0.024 |
| Folic acid, mg/kg | 2.3 |
| We verified betaine and choline concentrations in a water extract of the chow and measured 1300 mg betaine and 1350 mg choline per kg chow. | |

| SUPPLEMENTAL TABLE 2. Biosamples collected from the rats; storage and extraction conditions and markers measured in each tissue matrix. | | | |
| --- | --- | --- | --- |
| **Matrix** | **Preserved or stored** | **Extraction methods** | **Markers measured** |
| EDTA plasma | Stored at −80°C until analyses | None | Betaine, choline, dimethylglycine, taurine, liver markers, monoamine neurotransmitters (dopamine, serotonin, norepinephrine, 5-hydroxyindole acetic acid, 5-hydroxyindole acetylaldehyde) |
| EDTA plasma | Stored at −80°C until analyses | Homogenized in 1 N perchloric acid (20 µL/180 µL sample) | Monoamine neurotransmitters |
| Liver | Immediately snap-frozen, later thawed and extracted | Homogenized in 1 N acetic acid (10 µL/mg) | SAM and SAH |
| Hippocampus | Immediately snap-frozen and stored at −80°C, later thawed and extracted | Homogenized in 1 N acetic acid (10 µL/mg) | SAM and SAH, monoamines (dopamine, serotonin, norepinephrine, 5-hydroxyindole acetic acid, 5-hydroxyindole acetylaldehyde) |
| Prefrontal cortex | Immediately snap-frozen and stored at −80°C, later thawed and extracted | Homogenized in 1 N acetic acid (10 µL/mg) | SAM and SAH, monoamines (dopamine, serotonin, norepinephrine, 5-hydroxyindole acetic acid, 5-hydroxyindole acetylaldehyde) |
| Urine | Frozen at −80°C, later thawed and analyzed | None, only diluted 1:20 before measurements | Acetylcholine, betaine, choline, dimethylglycine |
| Stool | Frozen at −80°C, later thawed and extracted | Homogenized in 1 N acetic acid (10 µL/mg) | SAM |
|  |  | Homogenized in water (10 µL/mg) | Acetylcholine, betaine, choline, dimethylglycine, taurine |
| SAH, S-adenosylhomocysteine; SAM, S-adenosylmethionine. | | | |

| SUPPLEMENTAL TABLE 3. Performance of the methods used in the present paper according to the matrix used for method validation. | | |
| --- | --- | --- |
| **Marker** | **Matrix** | **Between-day coefficient of variation (CV%)** |
| Taurine (1) | EDTA plasma and urine | In plasma pool  3.9%  In urine pool 6.7% |
| Betaine, choline, and dimethylglycine (2) | EDTA plasma and urine | In plasma pool  Betaine: 5.8%  Choline: 8.4%  Dimethylglycine: 9.6%  In urine pool  Betaine: 4.8%  Choline: 7.9%  Dimethylglycine: 8.4% |
| Monoamines [dopamine, serotonin (5-HT), norepinephrine, 5-hydroxyindole acetic acid, 5-hydroxyindole acetylaldehyde] (In-house method) | EDTA plasma, prefrontal cortex, and hippocampus extracts | All < 6% |
| S-adenosylhomocysteine (SAH) and S-adenosylmethionine (SAM) (3) | EDTA plasma, and liver, prefrontal cortex, and hippocampus extracts | SAH: 8.3%  SAM: 10.1% |
| Published methods (1–3) were run on Acquity Ultra Performance LC system coupled to a MicroMass Quattro Premier XE tandem quadrupole mass spectrometer (Waters Corporation, Milford, MA, U.S.). | | |

| SUPPLEMENTAL TABLE 4. Concentrations of C1-metabolites and related compounds excreted in urine and stool extracts from Flinders Resistant Line (FRL) rats and Flinders Sensitive Line (FSL) rats treated with vehicle^1^ for 10 weeks. | | | | | |
| --- | --- | --- | --- | --- | --- |
|  | FRL (n = 8) | FSL (n = 7) | | | P^2^ |
| **Urine** | | | | | |
| Taurine, mmol/L | 4.49 ± 1.55 | | 4.42 ± 3.13 | 0.957 | |
| Betaine, µmol/L | 283 ± 91.0 | | 733 ± 1220 | 0.315 | |
| Choline, µmol/L | 110 ± 40.2 | | 48.1 ± 15.6 | 0.002 | |
| Dimethylglycine, µmol/L | 57.0 ± 37.1 | | 178 ± 121 | 0.004^3^ | |
| **Water stool extract** |  | |  |  | |
| Betaine, µmol/L^4^ | < LOD | | < LOD | - | |
| Choline, nmol/L | 3.10 ± 2.63 | | 2.37 ± 0.74 | 0.493 | |
| Dimethylglycine, µmol/L^4^ | < LOD | | < LOD | - | |
| SAM, nmol/g tissue^5^ | 33.4 ± 19.9 | | 34.5 ± 18.2 | 0.920 | |
| Data are presented as mean ± SD.  ^1^ Vehicle treatment consisted of xylitol, maize-derived maltodextrin, plum flavor, and malic acid.  ^2^ P-values were determined with the use of a one-way ANOVA test.  ^3^ P-values were determined with the use of a one-way ANOVA test performed on log-transformed data.  ^4^ The limits of detection (LOD) are 0.18–0.34 µmol/L for betaine and 0.12–0.13 µmol/L for dimethylglycine.  ^5^ SAM was measured in acidified water stool extracts (1 N acetic acid).  SAM, S-adenosylmethionine. | | | | | |

| SUPPLEMENTAL TABLE 5. Results of mood-related behavioural tests performed after 6–9 weeks of intervention with probiotics or vehicle. | | | | | |
| --- | --- | --- | --- | --- | --- |
|  | Vehicle^1^ | | 10^9^ CFU/d probiotics^2^ | | 10^10^ CFU/d probiotics^2^ |
|  | FRL | FSL | FSL | | FSL |
| Number | 8 | 7 | 7 | | 8 |
| **Novel Object Recognition (nonspatial memory)** | | | | | |
| Preference of novel/familiar object, % | 63.7 ± 19.0 | 55.5 ± 9.42 | 56.4 ± 16.6 | 58.6 ± 13.7 | |
| Total exploration time, s | 27.0 ± 10.0 | 35.2 ± 19.1 | 44.4 ± 24.7 | 52.3 ± 19.2 | |
| Discrimination index^3^ | 0.27 ± 0.38 | 0.11 ± 0.19 | 0.13 ± 0.33 | 0.17 ± 0.27 | |
| **Y-Maze (spatial memory)** | | | | | |
| Correct alternations, % | 67.3 ± 10.5 | 60.2 ± 18.2 | 59.8 ± 7.08 | | 66.4 ± 13.4 |
| **Elevated Plus Maze (anxiety)** | | | | | |
| Latency to enter open arms, s | 21.7 ± 18.4 | 13.9 ± 10.6 | 5.54 ± 4.91 | | 15.1 ± 14.0 |
| Time on open/closed arms, % | 50.4 ± 13.4 | 43.6 ± 24.5 | 30.4 ± 14.9 | | 50.7 ± 12.0 |
| Entries into open/closed arms, % | 44.9 ± 9.77 | 41.3 ± 18.6 | 32.4 ± 12.1 | | 46.4 ± 9.66 |
| **Social Interaction (social behavior)**^4^ | | | | | |
| Time spent sniffing, s | 95.3 ± 14.8 | 138 ± 48.2 | 135 ± 25.7 | | 132 ± 36.2 |
| **Pre-Forced Swim Test (depressive-like behavior)** | | | | | |
| Struggling, s | 105 ± 43.2 | 99.3 ± 20.7 | 126 ± 29.7 | | 128 ± 46.9 |
| Swimming, s | 93.8 ± 25.7^5^ | 56.4 ± 18.4 | 56.4 ± 21.4 | | 57.5 ± 30.1 |
| Immobility, s | 101 ± 26.2^5^ | 144 ± 17.7 | 117 ± 47.1 | | 115 ± 35.3 |
| **Open Field (locomotion)** | | | | | |
| Total distance, cm | 2884 ± 580^5^ | 3783 ± 550 | 3497 ± 422 | | 3879 ± 440 |
| Total velocity, cm/s | 9.63 ± 1.92^5^ | 12.7 ± 1.84 | 11.7 ± 1.42 | | 13.0 ± 1.49 |
| **Forced Swim Test (depressive-like behavior)** | | | | | |
| Struggling, s | 89.4 ± 31.1 | 77.1 ± 20.4 | 104 ± 29.4 | | 112 ± 59.5 |
| Swimming, s | 93.1 ± 23.1^5^ | 50.0 ± 20.8 | 56.4 ± 29.3 | | 40.6 ± 29.1 |
| Immobility, s | 118 ± 32.2^5^ | 173 ± 27.2 | 140 ± 39.9 | | 148 ± 54.1 |
| Data are presented as mean ± SD. The tests are shown according to the order of their performance. See Supplemental Figure 1 for a detailed experimental timeline of the behavioral tests performed.  ^1^ Vehicle treatment consisted of xylitol, maize-derived maltodextrin, plum flavor, and malic acid.  ^2^ Probiotic treatment additionally included *Lactobacillus helveticus* R0052 and *Bifidobacterium longum* R0175.  ^3^ The discrimination index was calculated as follows: [(Novel object exploration time − familiar object exploration time)/total exploration time].  ^4^ n = 3–4/group (rats tested in treatment-matched pairs)  ^5^ Significantly different from FSL vehicle, *p* ≤ 0.009 (one-way ANOVA)  CFU, colony-forming units; FRL, Flinders Resistant Line; FSL, Flinders Sensitive Line. | | | | | |

| SUPPLEMENTAL TABLE 6. Genes coding for enzymes related to C1-metabolism are present in *Lactobacillus helveticus* R0052 genome. | |
| --- | --- |
| **Enzyme** | **Function** |
| S-adenosylmethionine synthetase [EC [2.5.1.6](http://www.genome.jp/dbget-bin/www_bget?ec:2.5.1.6)] | Transfers an adenosyl from ATP and forms SAM from methionine |
| Betaine-homocysteine S-methyltransferase [EC [2.1.1.5](http://www.genome.jp/dbget-bin/www_bget?ec:2.1.1.5)] | Transfers methyl groups from betaine to homocysteine to produce methionine |
| Choline oxidase [EC [1.1.3.17](http://www.genome.jp/dbget-bin/www_bget?ec:1.1.3.17)] | Reduces choline to betaine |
| Choline dehydrogenase [EC [1.1.99.1](http://www.genome.jp/dbget-bin/www_bget?ec:1.1.99.1)] | Reduces choline to betaine |
| Methionine synthase [EC 2.1.1.13] | Synthesizes methionine by transferring a methyl group from folate to homocysteine |
|  |  |

| SUPPLEMENTAL TABLE 7. Stepwise multiple linear regression analysis applied to find predictors of plasma and prefrontal cortex concentrations of the main monoamines | | | | |
| --- | --- | --- | --- | --- |
| **Dependent variable** | **Independent variables with significant influence** | **Beta (95% CI)** | **p** | **Adjusted R^2^** |
| Plasma dopamine | Treatment  Plasma dimethylglycine  Plasma cystathionine  Plasma taurine  Hippocampus SAM | −4.63 (−6.16,−3.10)  −1.65 (−2.64,−0.66)  0.016 (0.007,0.025)  0.06 (0.01,0.11)  0.87 (0.36,1.39) | <0.001  0.003  0.002  0.035  0.003 | 0.84 |
| Plasma DOPAC | Hippocampus norepinephrine | −8.0 (−15.8,−0.2) | 0.045 | 0.18 |
| Plasma serotonin (5-HT) | Treatment  Prefrontal cortex SAH (nmol/g)  Plasma Hcy  Plasma taurine | −317 (−608,−26)  −927 (−1738,−116)  168 (80,255)  15.8 (9.6, 22.1) | 0.035  0.028  0.001  <0.001 | 0.73 |
| Plasma 5-HIAA | Hippocampus 5-HIAA  Prefrontal cortex 5-HIAA  Prefrontal cortex norepinephrine  Prefrontal cortex SAM, nmol/g  Treatment | 28.3 (14.7,42.0)  18.7 (6.0,31.4)  −28.2 (−35.6,−20.7)  −0.49 (−0.94,−0.03)  −1.9 (−3.0,−0.7) | 0.001  0.008  <0.001  0.039  0.004 | 0.95 |
| Plasma norepinephrine | Prefrontal cortex dopamine | 12.7 (0.22,25.1) | 0.047 | 0.18 |
| Prefrontal cortex dopamine | Prefrontal cortex DOPAC  Prefrontal cortex SAH  Plasma betaine  Plasma dimethylglycine  Plasma norepinephrine  Stool SAM  Body weight | 1.5 (1.4,1.6)  0.17 (0.06,0.29)  0.002 (0.001,0.003)  −0.05 (−0.07,−0.03)  0.004 (0.002,0.006)  −0.001 (−0.002,0.00)  −0.001 (−0.002,0.00) | <0.001  0.006  <0.001  <0.001  0.002  0.027  0.023 | 0.99 |
| Prefrontal cortex serotonin (5-HT) | Prefrontal cortex norepinephrine  Plasma dopamine  Stool choline  Plasma betaine  Plasma choline | 0.55 (0.38,0.72)  0.014 (0.009,0.019)  −0.035 (−0.060,−0.011)  −0.001 (−0.002,−0.001)  0.009 (0.003,0.015) | <0.001  <0.001  0.009  0.002  0.006 | 0.89 |
| The independent variables included in the model are (always excluding the dependent variable in question):  Treatment (1, vehicle; 2, low dose; 3, high dose); plasma (serotonin, 5-HIAA, DOPAC, dopamine, norepinephrine); plasma (dimethylglycine, betaine, choline, homocysteine, cystathionine, taurine, ALT), hippocampus and prefrontal cortex (SAM, SAH, dopamine, serotonin, DOPAC, 5-HIAA, norepinephrine); stool (SAM, choline); animal weight at week 10; and liver (SAH, SAM). | | | | |

Reference List

1. Awwad HM, Geisel J, Obeid R. Determination of trimethylamine, trimethylamine N-oxide, and taurine in human plasma and urine by UHPLC-MS/MS technique. J Chromatogr B Analyt Technol Biomed Life Sci 2016;1038:12-8.

2. Kirsch SH, Herrmann W, Rabagny Y, Obeid R. Quantification of acetylcholine, choline, betaine, and dimethylglycine in human plasma and urine using stable-isotope dilution ultra performance liquid chromatography-tandem mass spectrometry. J Chromatogr B Analyt Technol Biomed Life Sci 2010;878:3338-44.

3. Kirsch SH, Knapp JP, Geisel J, Herrmann W, Obeid R. Simultaneous quantification of S-adenosyl methionine and S-adenosyl homocysteine in human plasma by stable-isotope dilution ultra performance liquid chromatography tandem mass spectrometry. J Chromatogr B Analyt Technol Biomed Life Sci 2009;877:3865-70.
